# Monthly Record of Deaths in the City of Buffalo

**Published:** 1858-09

**Authors:** H. D. Garvin

**Affiliations:** Health Physician


ART. VI.—*Report of Mortality in Buffalo for the Month of July,* 1858. By H. D. Garvin, M. D., Health Physician.

| DISEASES. | No. | Males. | Females. | No Sex given. |
|---|---|---|---|---|
| Abdominal Tumor, | 1 | 1 | | |
| Abcess, | 1 | | 1 | |
| Accidental, | 6 | 5 | 1 | |
| Anemia, | 1 | | 1 | |
| Apoplexy, | 1 | | 1 | |
| Atrophia, | 1 | | 1 | |
| Caries of Spine, | 1 | 1 | | |
| Cholera Infantum, | 14 | 7 | 7 | |
| Cholera Morbus, | 1 | | 1 | |
| Convulsions, | 8 | 4 | 4 | |
| Coup de Soleil, | 1 | | 1 | |
| Delirium Tremens, | 4 | 3 | I | |
| Disease of Liver, | 1 | 1 | | |
| Disease of Brain, | 3 | 3 | | |
| Drowning, | 6 | 4 | 2 | |
| Dropsy, | 2 | 1 | 1 | |
| Dysentery, | 11 | 6 | 5 | |
| Enteritis, | 1 | | 1 | |
| Fever, Typhoid, | 3 | 3 | | |
| " Scarlet, | 4 | 3 | 1 | |
| " Puerperal, | 2 | | 2 | |
| " Congestive, | 1 | | 1 | |
| " Remittent, | 1 | | 1 | |
| Hæmorrhage, | 2 | 1 | 1 | |
| Heart Disease, | 1 | 1 | | |
| Hooping Cough, | 3 | 1 | 2 | |
| Hydrocephalus, | 3 | 2 | 1 | |
| Intemperance, | 1 | 1 | | |
| Marasmus, | 7 | 5 | 2 | |
| Old Age, | 3 | 2 | 1 | |
| Peritonitis, | 1 | 1 | | |
| Phthisis, | 7 | 4 | 3 | |
| Pneumonia, | 3 | 3 | | |
| Premature Birth, | 2 | 1 | 1 | |
| Puerperal Convulsions, | 1 | | 1 | |
| Rheumatism, | 1 | | 1 | |
| Salt Rheum, | 1 | | 1 | |
| Scirrhus, | 2 | 1 | 1 | |
| Scrofula, | 2 | 1 | 1 | |
| Still Born, | 4 | 2 | 2 | |
| Syphilis, | 1 | 1 | | |
| Teething, | 2 | 1 | 1 | |
| Thrush, | 2 | 1 | 1 | |
| Typhoid Pneumonia, | 1 | | 1 | |
| Ulceration of Bowels, | 1 | 1 | | |
| " " Stomach, | 1 | | 1 | |
| Unknown, | 7 | 3 | 4 | |
| Total, | 134 | | | |

### SEXES.

| | |
|---|---|
| Males, | 77 |
| Females, | 57 |
| Sex not given, | 0 |
| Total, | 134 |

### AGES.

| | | | |
|---|---|---|---|
| Still-born, | 4 | Between 20 years and 30 years, | 15 |
| 1 day, | 2 | " 30 " " 40 " | 10 |
| 1 day and 30 days, | 5 | " 40 " " 50 " | 9 |
| Between 1 month and 6 months, | 17 | " 50 " " 60 " | 6 |
| " 6 months and 12 " | 23 | " 60 " " 70 " | 6 |
| " 1 year " 3 years, | 15 | " 70 " " 80 " | 2 |
| " 3 " " 5 " | 5 | " 80 " " 90 " | 1 |
| " 5 " " 10 " | 4 | " 90 " " 100 " | 0 |
| " 10 " " 20 " | 6 | " 100 " | 0 |
| | 81 | | 49 |
| Ages not given, | 14 | | 134 |
| Total, | 126 | | |

### NATIVITIES.

| | | | |
|---|---|---|---|
| American, (colored, 2,) | 92 | Prussian, | 0 |
| German, | 14 | Italian, | 1 |
| Irish, | 18 | French, | 1 |
| English, | 4 | Scotch, | 0 |
| Canadian, | 2 | Switzerland, | 1 |
| Holland, | 0 | Country not given, | 0 |
| Total, | | | 134 |

---

*Contributions to Operative Surgery and Surgical Pathology.* By J. M. Carnochan, M. D., Prof. of Surgery in the New York Medical College; Surgeon-in-Chief to the State Emigrants Hospital, etc.

This is the first number of a series of contributions to operative surgery, consisting in a full account of some rare surgical cases. These are illustrated by admirable drawings from nature. The first number contains a case of "Amputation of the entire Lower Jaw," and cases of Elephantiasis Arabum treated by Ligature of the Femoral Artery.

Dr. Carnochan has published several remarkable cases of surgical disease, which he has operated upon with excellent success, and we are glad to see them presented to the profession in a collected form.

